# The association of chronotype and social jet lag with body composition in German students: The role of physical activity behaviour and the impact of the pandemic lockdown

**DOI:** 10.1371/journal.pone.0279620

**Published:** 2023-01-11

**Authors:** Bettina Krueger, Bianca Stutz, Nicole Jankovic, Ute Alexy, Anna Kilanowski, Lars Libuda, Anette E. Buyken

**Affiliations:** 1 Department of Sports and Health, Institute of Nutrition, Consumption and Health, Paderborn University, Paderborn, Germany; 2 Institute of Nutritional and Food Sciences (IEL), Bonn University, Bonn, Germany; 3 Institute of Epidemiology, Helmholtz Zentrum München - German Research Center for Environmental Health, Neuherberg, Germany; University of Lübeck: Universitat zu Lubeck, GERMANY

## Abstract

Young adults with a later chronotype are vulnerable for a discrepancy in sleep rhythm between work- and free days, called social jet lag (SJL). This study analysed (i) chronotype/SJL association with visceral fat/skeletal muscle mass, (ii) the attribution to physical activity behaviour, and (iii) chronotype-specific changes in physical activity behaviour in young adults during the Covid-19 pandemic lockdown. Chronotype and SJL were derived from the Munich-Chrono-Type-Questionnaire in 320 German students (age 18–25 years) from September 2019 to January 2020, 156 of these participated in an online follow-up survey in June 2020. Body composition was assessed by bioimpedance analysis at baseline. Multivariable linear regression analyses were used to relate chronotype/SJL to body composition; the contribution of self-reported physical activity was tested by mediation analysis. At baseline, a later chronotype and a larger SJL were associated with a higher visceral fat mass (P<0.05), this relation was notably mediated by the attention to physical activity (P<0.05). Chronotype (P = 0.02) but not SJL (P = 0.87) was inversely associated with skeletal muscle mass. During the pandemic lockdown, chronotype hardly changed, but SJL was reduced. Timing and physical activity behaviour remained in most participants and changes were unrelated to chronotype (all P>0.07). A later chronotype/higher SJL may increase the risk of a higher visceral fat mass even in this relatively healthy sample, which may be partly due to their physical activity behaviour. Despite a reduction in SJL during the pandemic lockdown, later chronotypes did not change their physical activity behaviour more than earlier chronotypes.

## Introduction

The chronotype is a biological construct describing the inter-individual preference of individuals for sleep and awake times [1]. Social circumstances such as work time schedules or school and university start times may lead to a misalignment between the “inner clock” (i.e. the chronotype) and the “social clock”, coined “social jet lag (SJL)”. This misalignment may contribute to an increased risk for obesity [2], a risk potentially already emerging during adolescence and young adulthood, when chronotype is generally delayed [3]. However, since body weight or body mass index (BMI) are known to be crude measures of the metabolically relevant visceral fat only [4], additional evidence is needed whether a higher visceral fat mass or lower skeletal mass are chronotype-specific in young adults (aim i). So far, only few data are available on parameters other than BMI and with focus on young adults. One study reported that evening types (later chronotypes) have a higher waist circumference and visceral fat mass compared to morning types independently of sex, age, BMI, physical activity, and adherence to a Mediterranean diet [5]. However, the mean age of those participants was approximately 50 years. In contrast, another study reported that neither chronotype nor SJL was associated with body composition outcomes such as percentage of body fat, waist-to-hip ratio, and waist-to-height ratio among young adults aged 21–35 years, but associations between sleep quality and body composition varied by chronotype [6].

Individuals with a later chronotype exhibit a preference for sedentary behaviour [7,8]. Therefore, it remains to be clarified whether lower levels of physical activity and/or a more sedentary lifestyle are implicated in the more adverse body composition among young adults with a later chronotype (see aim (ii)). This is conceptually plausible since both are established risk factors for overweight or obesity [9,10] and physical activity pattern appear to be synchronized with the inner clock [11]. Finally, timing seems to affect performance among athletes in a chronotype-specific manner: Earlier chronotypes perform better at earlier daytimes [11] and late chronotypes perform better in the evening [12].

Interesting insights could come from the exceptional conditions associated with the lockdown measures established due to the Covid-19 pandemic in spring 2020 in many countries [13]. In many people this caused a break from normal daily routines and might have affected circadian rhythms. Indeed, the Global Chrono Corona Survey (GCCS), which summarized studies from 40 countries assessing sleep-wake times before and during social restrictions [14], revealed that SJL decreased on average by 30 minutes whereas chronotype remained stable. This was also confirmed for German students [15]. Yet, lockdown associated changes in students’ physical activity and sedentary behaviour observed in Italy, Canada, Spain and Germany are mixed: While some studies reported a decrease in physical activity and an increase in sedentary behaviour [16–19] others reported increases in physical activity levels [20,21]. Thus far, only one study by Korman et al has examined whether the individual chronotype and physical activity during lockdown are interrelated. (see aim (iii)). In that study, persons with a later chronotype (mid-sleep time) reported a larger self-rated decrease in physical activity [22].

Hence, this study analysed (i) whether chronotype or SJL associate with skeletal muscle mass or visceral fat mass, (ii) whether this may be attributable to physical activity behaviour, and (iii) whether the lockdown resulted in chronotype-specific changes in physical activity behaviour in young adults.

## Materials and methods

### Study design

The Chronotype and Nutrition (ChroNu) study addresses the association of chronotype and social jetlag with body composition in students. From September 2019 to January 2020, students from all faculties of Paderborn University aged 18–25 years and BMI >18.5kg/m^2^ were invited to participate. Exclusion criteria were: pregnancy or lactation, shift work in the past 3 months, crossing of >1 time zone in the previous 3 months, intake of sleep affecting medications such as antidepressants and sedatives. Students filled in questionnaires on general characteristics, living conditions, and smoking behaviour as well as their time schedules at university, jobs and time spent outdoors. Questions on physical activity behaviour were adapted from the DEGS survey (“Studie zur Gesundheit Erwachsener”) [23] and addressed frequency and duration of moderate-to-vigorous physical activity (“How many days a week are you physically active enough to break a sweat or get out of breath?”) as well as exercise frequency and type of sport during the past four weeks. Exercise type was categorized into endurance, strength, relaxation, and “others” (e.g. football, basketball, horse riding, and climbing); all ball games, horse riding, etc. were included in the category endurance whereas e.g. climbing was regarded as strength training. Timing of exercise (morning 6:00–11:00, midday 11:00–14:00, afternoon 14:00–18:00, evening 18:00–21:00, night 21:00–6:00) was inquired separately for workdays and free days. Self-reports on the attention to physical activity (i.e. answers to the question: “How much attention do you pay to physical activity”) could be rated as “very important”, “important”, “partly”, or “not at all”. This variable reflects a cognitive variable than a variable of physical activity. Sedentary behaviour was assessed by the time spent on screens assessed separately for work- and free days.

The study was approved by the Ethics Committee at Paderborn University and registered at the clinical trial registration (clinical trial gov ID NCT04302922). Informed consent was obtained from all participants prior to participation.

Due to the COVID-19 pandemic, lockdown restrictions were set up in Germany from mid-March 2020 to June 2020 [24], involving the closure of schools, universities, kindergartens and non-system-relevant businesses. Paderborn University started the summer term at the end of April implementing online teaching and self-guided learning at home. In June 2020, i.e., at the end of pandemic lockdown all participants were re-contacted and asked to fill in an online survey via the university-internally used software application Redcap (Research Electronic Data Capture, developed by Vanderbilt University, USA [25] addressing the same questions as at baseline.

### Participants

Overall, 327 students (18 to 25 years, 58% females) were included at baseline. Six persons had to be excluded because they reported to work night shifts and one person reported to take antidepressant medication. Hence, 320 participants were included in the baseline cross-sectional analysis (aims i and ii).

A total of 192 students provided online feedback on their circadian behaviours during the COVID-19 pandemic lockdown. Of these, 27 were excluded due to incomplete surveys, 3 of the 7 excluded from the baseline assessment responded the online feedback and had to be excluded subsequently, 4 surveys were not considered because of double submission and 2 because of invalid identification numbers.

### Chronotype

Chronotype was assessed by the Munich Chrono-Type Questionnaire (MCTQ). The MCTQ correlates individual circadian behaviour with social circumstances by capturing bedtimes and wake up times on work and free days, respectively [26]. Chronotype is defined as midpoint of sleep on free days corrected for sleep debt on work days (MSFsc) and SJL is calculated as the difference between midpoint of sleep during work days and free days [27].

### Anthropometric data

Body composition was measured by bioimpedance (BIA) (SECA mBCA 515), a validated method [28] estimating body fat mass (%), visceral fat mass (L), and skeletal mass (kg). In addition, SECA mBCA also measures body weight (kg) and height (m) (seca 287 dp), allowing for the calculation of BMI. Participants were measured barefoot and while wearing underwear only. Waist circumference measures were taken according to standard procedures [29].

### Statistical analysis

All statistical analyses were performed using SAS procedures (version 9.4; Cary, NC, USA). P values of <0.05 were considered statistically significant. Interaction analyses showed no interaction of the investigated association with sex, thus data from both sexes were pooled for analysis.

Smoking behaviour was categorised as any smoking (occasionally or regularly) yes/no. Living conditions were categorised as living in shared apartments yes/no. Screen time behaviour was summarized as spending more than 4 hours with computer, smartphones, TV, etc. (yes/no). A high frequency of physical activity was defined as “4 or more days per week” (yes/no), a high duration of physical activity as “more than 30 to 60 minutes” (yes/no). A physical activity of 150 min/week or more was defined as being in accordance with the WHO recommendation (yes/no). A high exercise frequency was defined as ≥2 hours per week (yes/no). Type of exercise was categorized in endurance (yes/no) or strength (yes/no). The attention to physical activity was categorised in “high” (yes/no) if the question “How much attention do you pay to physical activity”, was answered by “very important” or “important”. Timing of sport was categorized as “early” combining time frames in the morning and midday (6:00–11:00, 11:00–14:00) and “late” combining time frames in the afternoon, evening, and night (14:00–18:00, 18:00–21:00, 21:00–6:00).

Participant characteristics are presented as mean (+/-SD), median (Q1, Q3) or percentages. Physical activity behaviour is also presented by tertiles of chronotype and trends across tertiles were tested by the Mantel-Hanszel Chi-Square test.

Multivariable linear regression analyses were used to relate chronotype or SJL to body composition (aim i). To achieve a normal distribution of the outcome variables, the values were transformed 1/x. Crude models adjust for sex and age. The final models adjust for covariates that altered the predictor-outcome association by more than 10% [30] or were statistically significant (P≤ 0.05). Potential confounding covariates considered for inclusion were height, smoking, jobs, living conditions, sedentary variables such as screen time and physical activity behaviour such as time spend outdoors, frequency and duration of physical activity, frequency and types of sports as well as timing of sports on work- and free days and the attention to physical activity.

Retransformed results from regression analysis are presented as adjusted least-square means (95% confidence interval (CI)) by sex-specific tertiles; p-values are obtained from models using the exposure as continuous variables.

Variables of physical activity were considered as mediator in an additional mediation analysis (aim ii). The percentages of the association of chronotype or SJL with visceral fat mass or skeletal muscle mass attributable to the respective physical activity variables were calculated by the CAUSALMED procedure in SAS. The analysis was adjusted for the same covariates as in the final model of the multivariable linear regression analysis.

Changes in categorical parameters such as living conditions, physical activity and sedentary behaviour during the Covid-19 pandemic were evaluated by subtracting the value of the category at baseline from the value of category during lockdown. In cases where a lower value indicated a higher frequency/intensity, the value in lockdown was subtracted from the baseline vales, to ensure that a negative value unanimously represents a decrease in the categories referring to frequency/intensity. To assess changes at follow-up, a simple change variable was constructed with 3 levels: "increase" (i.e., increase from baseline to follow-up by at least one category), "no change" (i.e., same category at follow-up and baseline), or "decrease" (i.e., decrease from baseline to follow-up by at least one category). Relations between changes in categories of physical and exercise variables (increase/ no change/ decrease) and tertiles of chronotype were tested by Mantel-Hanszel Chi-Square test (aim iii).

## Results

The ChroNu study included 184 women and 136 men. Participants of the ChroNu cohort were on average 22.8 years old and had a mean BMI of 23.1 kg/m^2^ (Table 1). Median mid-sleep point—corrected for sleep debt during the week—(i.e. the median chronotype) was at 4:35 o’clock and the median SJL amounted to 1:07 h:min.

**Table 1:** Characteristics of German university students aged 18–25 years at baseline assessment of the ChroNu study (n = 320).

	Characteristics
Sex n (%) females	184 (57.5)
Age (years)	22.8 ± 1.9
BMI (kg/m^2^)	23.1 ± 3.0
Smokers n (%)	32 (10)
Job n (%)	121 (38)
*Body composition*	
Visceral fat mass (kg)	
Males	0.68 (0.30; 1.15)
Females	0.37 (0.19; 0.58)
Skeletal muscle mass (kg)	
Males	32.0 (29.5; 35.0)
Females	20.3 (18.8; 22.2)
FMI (kg/m^2^)	
Males	3.8 (2.8; 5.2)
Females	5.8 (4.8; 7.4)
FFMI (kg/m^2^)	
Males	19.7 (18.6; 21.0)
Females	16.1 (15.4; 17.1)
*Chronotype and social jet lag*	
Chronotype (o’clock).	4:35 (3:50; 5:18)
Social jet lag (h:mm)	1:07 (0:42; 1:45)
*Living conditions n (%)*	
With parents	75 (24)
Shared apartments	150 (47)
couple	37 (12)
alone	58 (18)
Own family	0 (0)
Time outdoors workdays (h:mm)	1:00 (1:00; 2:00)
Time outdoors free days (h:mm)	2:00 (1:00; 3:00)
*Sedentary behavior n (%)*	
Screen time >4 h/day on workdays	167 (52)
Screen time >4 h/day on free days	132 (42)
*Physical activity (PA) behavior n (%)*	
PA frequency* ≥ 4 days/week	69 (22)
PA duration ≥ 30 min/unit	269 (84)
PA ≥150 min/week **	60 (19)
Exercise frequency ≥2 h/week	185 (58)
Exercise type endurance	242 (76)
Exercise type strength	192 (60)
*Exercise timing* ***	
on workdays n (%)	
Early (6 a.m. to 2 p.m.)	97 (30)
Late (after 2 p.m.)	268 (84)
on free days n (%)	
Early (6 a.m. to 2 p.m.)	144 (45)
Late (after 2 p.m.)	227 (71)
*Attention to physical activity n (%)*: “important or very important”	171 (54)

Abbreviations: BMI—body mass index, FMI—fat mass index, FFMI—fat free mass index, n—sample size, smoker (yes/no), job (yes/no), PA—physical activity.

Data are frequencies, mean (SD) or medians (Q1; Q3).

Chronotype refers to the clock time of the mid-point of sleep, social jetlag refers to time (hours:Minutes).

* physical activity refers to all kind of exhaustive physical activity including exercise.

** high physical activity means > 150 minutes/week according to the WHO recommendations (https://www.who.int/news-room/fact-sheets/detail/physical-activity).

***multiple answers were possible.

**Fig 1:**
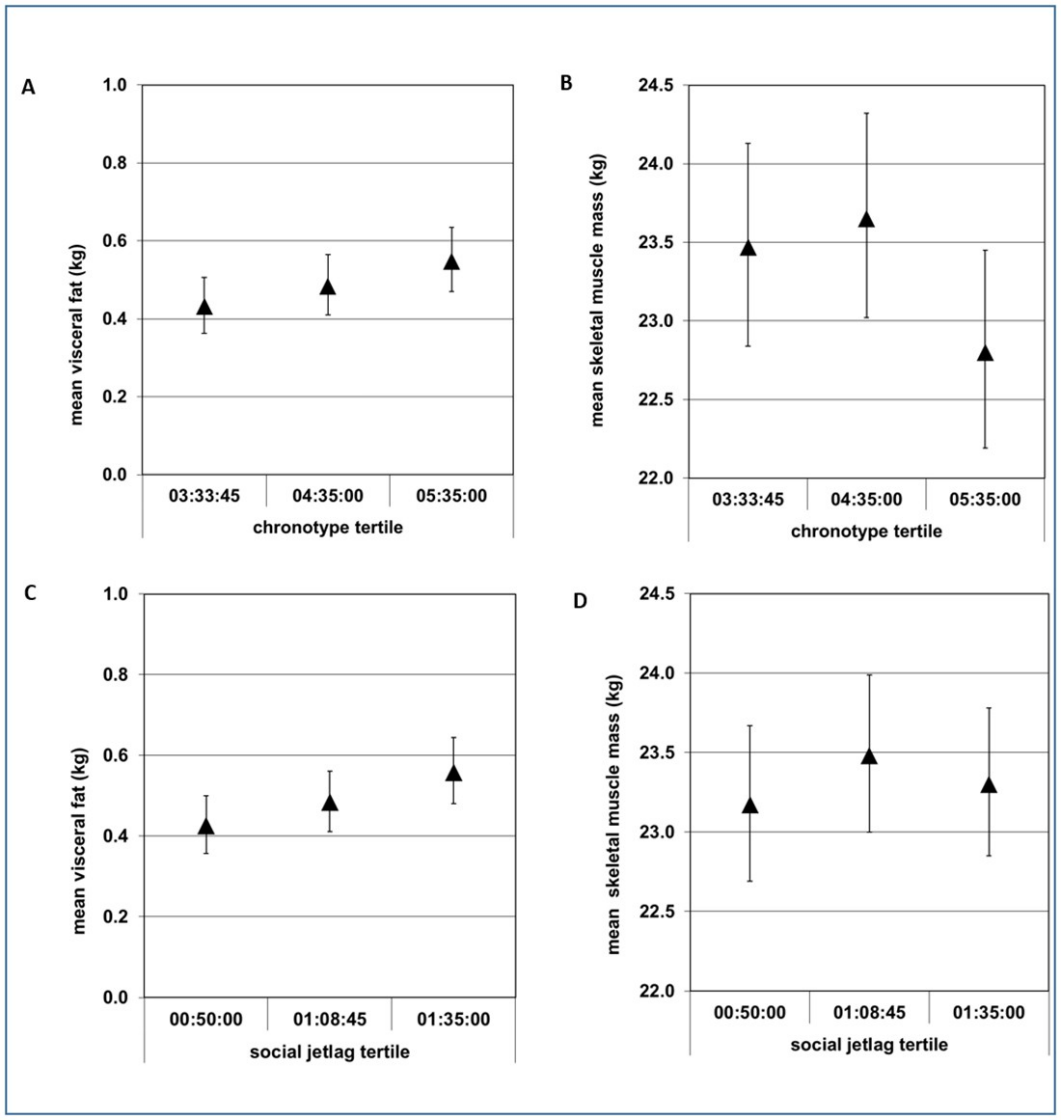
Least square means of body composition measures (95% confidence intervals) by chronotype or social jetlag tertiles: Upper panels: (A) Visceral fat mass (kg) or (B) skeletal muscle mass by tertiles of chronotype. Time in each tertile represents the mean midpoint of sleep—corrected for sleep debt during the week—in the respective tertile. P for trend = 0.048 in (A) and = 0.021 in (B). Lower Panels: (C) visceral fat mass (kg) or (D) skeletal muscle mass by tertiles of SJL. Time in each tertile represents the mean SJL. P for trend = 0.011 in (C) and = 0.869 in (D). Models in panel (A) and (C) are least-square means adjusted for age, sex, and living in shared apartments. Models in (B) and (D) are adjusted for age, sex and height. P-values refers to the value obtained from the multivariable regression model using chronotype or social jetlag as a continuous variable.

Regarding aim i, cross-sectional multivariable linear regression analyses revealed that a later chronotype and a larger SJL were related to a higher visceral fat mass (Fig 1; Table 2). Conversely, chronotype but not social jet lag was associated with a lower skeletal muscle mass (Fig 1, Table 2).

**Table 2:** Multivariable regression analysis of the association of chronotype/social jet lag with visceral fat/skeletal muscle mass mass (kg).

	Chronotype (MSF_sc_)				Social jet lag (SJL)			
	Tertile 1 (95% CI)	tertile 2 (95% CI)	tertile 3 (95% CI)	P for trend*	tertile 1 (95% CI)	tertile 2 (95% CI)	tertile 3 (95% CI)	P for trend*
**crude model**								
visceral fat mass	0.44 (0.37, 0.51)	0.49 (0.41, 0.57)	0.55 (0.47, 0.63)	0.085	0.42 (0.36, 0.50)	0.49 (0.42, 0.57)	0.56 (0.48, 0.65)	0.005
skeletal muscle mass	23.8 (23.1, 24.4)	23.80 (23.1, 24.4)	23.45 (22.9, 24.1)	0.440	23.44 (22.8, 24.1)	24.0 (23.4, 24.7)	23.56 (23.0, 24.2)	0.833
**adjusted Model**								
visceral fat mass^a^	0.43 (0.36, 0.51)	0.48 (0.41, 0.56)	0.55 (0.47, 0.63)	0.048	0.43 (0.36, 0.50)	0.48 (0.41, 0.57)	0.56 (0.48, 0.65)	0.011
skeletal muscle mass^b^	23.47 (23.0, 24.0)	23.65 (23.2, 24.2)	22.81 (22.4, 23.3)	0.021	23.17 (22.7, 23.7)	23.49 (23.0, 24.9)	23.3 (22.8, 23.8)	0.869

Data are least square means of visceral fat mass (kg) by tertiles of chronotype or social jet lag.

Abbreviations: MSF_sc_—Midpoint of sleep corrected, CI—confidence interval.

Crude model adjusted for age and sex.

^a^model adjusted for age, sex, and living in shared apartments.

^b^model adjusted for age, sex, height.

*P for trend refers to the value obtained from the multivariable regression model using MSF_sc_ or social jet lag as continuous variable.

Regarding aim ii, the mediation analysis revealed that only participants’ attention to physical activity contributed to the observed association of chronotype/SJL with visceral fat/skeletal muscle mass (Table 3). All other physical behaviour variables did not explain significant percentages of the observed associations (all p>0.06, see Table 3 for the most relevant variables). Similarly, sedentary behaviour was not relevant for this association (all p>0,1).

**Table 3:** Mediation analysis of the relevance of selected physical activity variables (potential mediators) for the association of chronotype/social jet lag with visceral fat / skeletal muscle mass (kg) among participants of the baseline survey (n = 320).

Exposure variable	Outcome variable	Potential mediator variable	Effect size*** (95% CI)	Mediated percentage	P
Chronotype*	visceral fat mass	attention to PA	-0.01403 (-0,02; -0,006)	72%	0.0459
Chronotype*	visceral fat mass	Exercise frequency ≥2h/week	-0.00555 (-0.01; -0.0002)	28.5%	0.1045
Chronotype*	visceral fat mass	PA ≥150 min/week	-0.00307 (-0.008; 0.002)	15.8%	0.2255
Chronotype*	visceral fat mass	screen time >4h/free days	-0.00393 (-0.008; 0.001)	20.2%	0.1778
Chronotype*	visceral fat mass	screen time >4h/work days	-0.00043 (-0.0004; -0.002)	2.2%	0.5448
Social jetlag*	visceral fat mass	attention to PA	-0.00958 (-0.02; -0.001)	31%	0.0422
Social jetlag*	visceral fat mass	Exercise frequency ≥2h/week	-0.00467 (-0.01; 0.002)	15.1%	0.1530
Social jetlag*	visceral fat mass	PA ≥150 min/week	-0.00293 (-0.009; 0.003)	9.5%	0.3045
Social jetlag*	visceral fat mass	screen time >4h/free days	-0.00182 (-0.005; 0.002)	5.8%	0.2965
Social jetlag*	visceral fat mass	screen time >4h/work days	-0.00039 (-0.002; 0.001)	1.3%	0.6177
Chronotype**	skeletal muscle mass	attention to PA	0.000281 (0.0001; 0.0005)	52.5%	0.0314
Chronotype**	skeletal muscle mass	Exercise frequency ≥2h/week	0.000139 (0.000001; 0.0003)	26%	0.0762
Chronotype**	skeletal muscle mass	PA ≥150 min/week	0.000075 (-0.00004; 0.0002)	14%	0.2217
Chronotype**	skeletal muscle mass	screen time >4h/free days	-0.00001 (-0.0001; 0.00008)	-2%	0.8201
Chronotype**	skeletal muscle mass	screen time >4h/work days	0.000012 (-0.00003; 0.00005)	2.2%	0.5702

Abbreviations: PA—physical activity, CI—confidence interval.

*model adjusted for age, sex, and living in shared apartments.

**model adjusted for age, sex and height.

***Effect size based on variables transformed 1/x and is the natural indirect effect of the mentioned physical activity mediator on the association between chronotype/SJL on visceral fat mass/skeletal mass.

During the COVID-19 pandemic lockdown, the median chronotype hardly changed in comparison to baseline assessment before lockdown (-0:05 h:mm), specifically earlier chronotypes (tertile 1) became slightly later and later chronotypes (tertile 3) became earlier (Table 4). By contrast, SJL was reduced by approximately 0:25 h:mm, with the largest decrease among later chronotypes (Table 4). Time spent outside increased by approximately 10 to 25 minutes on free days, but not on workdays (Table 4).

**Table 4:** Changes in chronotype, social jetlag and life factors from baseline (Oct 2019-Jan 2020) to follow-up (June 2021) by tertiles of chronotype at baseline (n = 156).

		**Tertile 1**	**Tertile 2**	**Tertile 3**	
*Differences in*					
Chronotype (h:mm)		0:03 (-0:23; 0:48)	-0:03 (-0:30; 0:23)	-0:38 (-1:04; 0:20)	
Social jet lag (h:mm)		-0:15 (-0:43; -0:10)	-0:23 (-1:00; 0:08)	-0:49 (-1:34; -0:18)	
Time outdoors workdays (h:mm)		0:00 (-0:30; 1:00)	0:00 (-0:40; 0:40)	0:15 (-0:15; 1:00)	
Time outdoors free days (h:mm)		0:10 (-0:15; 1:45)	0:30 (0:00; 1:30)	0:25 (0:00; 1:40)	
	**all**	**Tertile 1**	**Tertile 2**	**Tertile 3**	**P for trend**
*Living conditions n (%)*					
living in shared apartments^a^					
• not anymore at follow-up	5 (3)	2 (1)	0 (0)	3 (1.9)	0.7998
• unchanged	135 (87)	45 (29)	47 (30)	43 (28)	
• at follow-up only	16 (10)	4 (3)	6 (4)	6 (4)	
*Sedentary behavior n (%)*					
Screen time on workdays^b^^,^^c^					
• decrease at follow-up	23 (15)	7 (5)	8 (5)	8 (5)	0.9643
• unchanged	59 (38)	21 (14)	18 (12)	20 (13)	
• increase at follow-up	74 (48)	23 (15)	27 (17)	24 (16)	
Screen time on free days^b^^,^^c^					
• decrease at follow-up	43 (28)	11 (7)	20 (13)	12 (8)	0.1925
• unchanged	67 (43)	18 (12)	22 (14)	27 (18)	
• increase at follow-up	46 (30)	22 (14)	11 (7)	13 (9)	
*Physical activity behavior n (%)*					
PA frequency^c^^,^^d^					
• decrease at follow-up	40 (26)	17 (11)	13 (8)	10 (7)	0.2481
• unchanged	40 (26)	11 (7)	13 (8)	16 (10)	
• increase at follow-up only	76 (49)	23 (15)	27 (17)	26 (17)	
PA duration^c^^,^^e^					
• decrease at follow-up	47 (30)	13 (8)	16 (10)	18 (12)	0.9916
• unchanged	74 (48)	30 (19)	23 (15)	21 (14)	
• increase at follow-up	35 (23)	8 (5)	14 (9)	13 (8)	
PA ≥150 min/week^c^^,^^f^					
• not any more at follow-up	8 (5)	3 (2)	5 (3)	0 (0)	0.7426
• unchanged	104 (67)	32 (21)	35 (23)	37 (24)	
• at follow-up only	44 (28)	16 (10)	13 (8)	15 (10)	
Exercise frequency^c^^,^^g^					
• decrease at follow-up	41 (26)	13 (8)	14 (9)	14 (9)	0.6982
• unchanged	68 (44)	25 (16)	22 (14)	21 (14)	
• increase at follow-up	47 (30)	13 (8)	17 (11)	17 811)	
Exercise type: endurance^c^^,^^h^					
• not any more at follow-up	16 (10)	7 (5)	4 (3)	5 (3)	0.5544
• unchanged	118 (76)	36 (23)	44 (28)	38 (25)	
• at follow-up only	22 (14)	8 (5)	5 (39	9 (6)	
Exercise type: strength^c^^,^^h^					
• not any more at follow-up	34 (22)	9 (6)	12 (8)	13 (8)	0.2103
• unchanged	95 (61)	31 (20)	32 (21)	32 (21)	
• at follow-up only	27 (17)	11 (22)	9 (17)	7 (14)	
*Exercise timing* ^c^ ^,^ ^i^					
Exercise on workdays n (%)*					
Early (6 a.m. to 2 p.m.)					
• not any more at follow-up	18 (12)	9 (6)	4 (3)	5 (3)	0.6012
• unchanged	108 (69)	31 (20)	40 (26)	37 (24)	
• at follow-up only	30 (19)	11 (7)	9 (6)	10 (7)	
Late (after 2 p.m.)					
• not any more at follow-up	14 (9)	5 (3)	4 (3)	5 (3)	0.6561
• unchanged	125 (80)	39 (25)	44 (28)	42 (27)	
• at follow-up only	17 (11)	7 (5)	5 (3)	5 (3)	
Exercise on free days n (%)*					
Early (6 a.m. to 2 p.m.)					
• not any more at follow-up	23 (15)	7 (5)	3 (2)	13 (8)	0.5001
• unchanged	101 (65)	35 (23)	38 (25)	28 (18)	
• at follow-up only	32 (21)	9 (6)	12 (8)	11 (7)	
Late (after 2 p.m.)					
• not any more at follow-up	29 (19)	10 (7)	10 (7)	9 (6)	0.5222
• unchanged	97 (62)	28 (18)	34 (22)	35 (23)	
• at follow-up only	30 (19)	13 (8)	9 (6)	8 (5)	
*Attention to PA*: n (%)^c^^,^^j^					
• decrease at follow-up	29 (19)	11 (7)	8 (5)	10 (7)	0.6910
• unchanged	84 (54)	29 (19)	29 (17)	28 (18)	
• increase at follow-up	43 (28)	11 (7)	18 (12)	14 (9)	

Abbreviations: PA—physical activity. Data are frequencies or medians (Q1; Q3); n, sample size, (%) percent of this tertile.

MSFsc, midpoint of sleep corrected.

Maentel-Hanzsel-Square test was performed for trend analysis for categorical variables.

*multiple answers were possible.

^a^Living conditions: “Not anymore at follow-up” refers to a change from living in shared apartments towards other living conditions, “at follow-up only” refers to a change from living in any other conditions towards living in shared apartments.

^b^ Screen time on workdays inquired how many hours were spent on screens (computer, tablets etc.) during the week. This was inquired in 7 categories as follows: Not at all, max. 30 minutes/day, 30–60 min, 1–2 hours, 3–4 hours, 4–6 hours, >6 hours/day.

^c^To assess changes at follow-up, distribution across the categories were compared and reduced to a simple change variable with 3 levels as follows: "Increase" (i.e., increase from baseline to follow-up by at least one category), "no change" (i.e., same category at follow-up and baseline) or "decrease" (i.e., decrease from baseline to follow-up by at least one category).

^d^ PA frequency inquired how many days participants were physically active (all exhaustive activities, including also activities such as cycling to University) per week. This was inquired in 8 categories as follows: Not at all, 1 day, 2, 3, 4, 5, 6, 7 days.

^e^ PA duration inquired how many minutes one unit of physical activity last. This was inquired in 4 categories as follows: Less than 10 min, 10–30 min, 30–60 min, more than 60 min.

^f^ high physical activity is equivalent to > 150 minutes/week according to the WHO recommendations. “Not any more at follow-up” refers to missing the WHO recommendations during the lockdown and “at follow-up only” refers to meeting the WHO recommendations during the lockdown.

^g^ Exercise frequency inquired how many hours were spent on exercises per week. This was inquired in 5 categories as follows: Not at all, less than 1 hour per week, regularly 1–2 hours, regularly 2–4 hours, >4 hours per week.

^h^exercise type “endurance” or “strength” refers to whether the exercise is categorized to endurance or strength. “Not any more at follow-up” refers to a change towards the other exercise type (endurance or strength) or not exercising at all during the lockdown and “at follow-up only” refers to a change towards this category of exercise.

^i^exercise timing “refers to whether time of exercises were before 2 p.m. (“early”) or after (“after”). “not any more at follow-up” refers to not exercising during this time frame of the day during the lockdown and “at follow-up only” refers to exercising during this time frame of the day during the lockdown.

^j^Attention to physical activity inquired how much attention was paid to physical activity. This was inquired in 5 categories as follows: Not at all, little, partly, important, very important.

Regarding aim (iii), all changes in physical activity behaviour were unrelated to chronotype (Table 4). During lockdown, approximately 50% of participants spent more time on screens, particularly on workdays. On the other hand, approximately 50% of participants increased the frequency, but only 35% increased the duration of physical activity per day. In consequence, a more students met the recommendation of the WHO to be physically active for at least 150 min/week. Frequency, type or timing of exercise hardly changed.

Students participating in the online survey during the lockdown and those not participating had similar characteristics (S1 Data), yet only 21% of non-participants achieved the WHO recommendations for physical activity at baseline in comparison to 85% of the participants.

## Discussion

Our data show that a later chronotype and a higher social jet lag are cross-sectionally associated with a higher visceral fat mass, but only a later chronotype is related to a lower skeletal muscle mass among German students. These adverse associations appear to be partly attributable a lower personal relevance for physical activity (or attention to physical activity). The lockdown in spring 2020 during the Covid-19 pandemic seemed to allow for a life more in accordance with the inner clock since a decrease in social jetlag was seen particularly among those with a later chronotype. However, changes in physical activity and sedentary behaviour were not chronotype-specific.

A misalignment of chronotype and social schedules may contribute to weight gain [31] and a higher BMI [32]. In particular, SJL was associated with higher BMI and higher fat mass, higher waist circumference and a higher likelihood for obesity [33]. Our study extends these findings confirming that chronotype and SJL are indeed selectively relevant to visceral fat mass and—albeit to a lesser extent—to skeletal muscle mass in young adults. Our findings are in line with the above mentioned previous study among middle aged adults (on average 50 years of age) suggesting that a more preferred eveningness was associated with a higher waist circumference and a higher visceral adipose tissue but not with subcutaneous adipose tissue [5]. To our knowledge, reports on the association between chronotype and visceral fat mass are currently limited to middle-aged samples [5,34] and is has not been reported whether this also applies to young adults with a normal BMI, yet comparably late chronotype.” Of note, our study suggests that selective adverse associations may already be discernible at a young age in a relatively healthy sample, which calls for a consideration of chronotype in future public health strategies. The novelty of our study is also that we emphasized the role of physical activity in this context.

In our analysis, attention to physical activity but not sedentary behaviour was relevant for the association between chronotype/SJL and visceral fat mass nor chronotype and skeletal muscle mass. Our data demonstrate that the individual attitude towards physical activity mediated the association between chronotype/SJL and visceral fat mass. Whilst those paying more attention to physical activity also exercised more, and were more likely to meet the WHO recommendation, those variables did not explain the association to a significant extent. Of note, our mediation analysis showed that the variable attention to physical activity explained as much as approximately 72% of the association between chronotype and visceral fat mass. However, as effect estimates in causal mediation analyses were rather small, the estimated proportions of explained association have to be interpreted with caution. This behavioural trait might indicate that earlier chronotypes have a higher conscientiousness towards physical activity. Alternatively, this variable may capture a general trait related to a healthier lifestyle and/or a higher self-discipline which might be more common among earlier chronotypes [35–37]. A recent publication related personal traits to chronotype reporting that higher conscientiousness and lower openness to experience were predictors for an earlier chronotype whereas excitement-seeking and less self-disciplined persons were more likely to have a later chronotype [37]. Of note, our results do not preclude that other factors such as meal-timing, food choice etc. as shown by others [38] might also be relevant (or more relevant) to this association.

Lower levels of physical activity are an established behavioural risk factor for increased visceral fat mass [9] and studies consistently reported lower levels of physical activity among persons with a later chronotype [35]. From a physiological point of view, it is interesting to note that e.g. body temperature is increased during the day and peaks in the afternoon whereas cortisol displays a peak in the morning [11,39]. These two factors may influence the optimal timing of maximal exercise performance since, indeed, body temperature and peak cortisol expression is delayed in later chronotypes compared to earlier chronotypes [35]. However, in our healthy cohort of students, later chronotypes followed their internal clock and exercised at later times of the day, which may explain why timing of physical activity did not emerge as relevant for the association of chronotype/SJL with visceral fat or skeletal muscle mass.

In studies available to date, physical activity was considered as a confounder variable in analyses on the association of chronotype with selective components of body composition such as BMI and waist-to-hip ratio in young adults [6,40]. None of these studies examined those variables as potential mediators on the association between chronotype and visceral fat or skeletal muscle mass yet. Failure to consider physical activity as a mediator rather than a confounder may however result in incorrect reports of null-associations between chronotype or SJL and anthropometric variables since physical activity may lie on the pathway between these exposure-outcome associations. In fact, in two recent studies among participants aged 21–60 years those with early and late chronotype did not differ in BMI [6,40], body fat, waist-to-height or waist-to-hip ratio [6] and these associations were further attenuated when adjusting for physical activity [6,40]. Hence, future studies should investigate the mediating role of physical activity behaviour rather than simply adjusting for it.

Showing that chronotype hardly changed during the lockdown our data underpin the proposal that chronotype is a biological construct which is mainly determined by age, sex, and genetics [27]. This observation was previously shown by a study conducted among German students [15] although recent studies observed larger changes in chronotype, sleep duration and SJL [22,41,42]. As our findings are in accordance with this other German study, this minor effect might be attributable to the lockdown conditions in Germany. On the other hand, SJL was decreased reflecting sleep-activity cycles more in accordance with the individual chronotype [15,42], as also seen in our study. In contrast to other countries, the lockdown terms in Germany allowed outdoor activities. We observed only minor reductions in outdoor light exposure and diverse adaptations of physical activity in our cohort whereas others reported larger reduction during social restrictions which is in line with the fact that our participants did not change their physical activity dramatically [22,43]. Lower levels of physical activity often go along with increased screen time. In our study at least half of our participants of our cohort also increased screen time. This may affect sleep time and hence chronotype: in fact a recent study reported an association between increased screen time and prolonged sleep latency during lockdown [44]. Under the assumption that life against the individual chronotype might adversely affect the motivation to be physically active, one could expect that the lockdown conditions allowed later chronotypes to increase their physical activity to a larger extent than earlier chronotypes. Yet, a recent study found that persons with a later chronotype reported more pronounced decreases in their physical activity, however these changes (increases/decreases) were self-rated using a 5-item Likert scale only [22]. By contrast in our study self-reported increases were seen for different parameters of physical activity, which is in line with other previous studies [14,42], yet these changes were not chronotype dependent. Hence, despite a larger reduction in SJL among later chronotypes, this was not accompanied by larger increases in physical activity behaviour. A reason for this might be that the Covid-19 measures themselves also restricted exercise opportunities, e.g., closure of fitness centres and sports clubs and daily routine physical activities such as cycling to university were no longer necessary. However, since locations of physical activity and exercise were not inquired by our questionnaires we cannot provide insight on this. Alternatively, students may have continued their usual time schedules due to online lectures and this may have extended to their physical activity behaviour or its timing. Of note, measures were toned down again towards the end of June 2020 in Germany, e.g., using the library or selected practical classes was possible again at Paderborn University. Yet, our questionnaires referred to the past 4 weeks, i.e., the last weeks of May and June.

Limitations of the study include the fact that only 156 out of 320 participants of the cohort at baseline participated in the follow-up online survey. However, this subgroup did not differ in chronotype or SJL, and body composition in comparison to those who did not participate. Furthermore, we used the validated DEGS questions to assess exercise behaviour [23], yet these questions referred to exercise for predefined time spans rather than inquiring exact times for exercise. Finally, baseline and follow-up refer to different seasons (i.e. autumn/winter times and early summer, respectively), yet sleep pattern [45] and activity [46] pattern might be season-dependent. We cannot rule out that a change in chronotype is masked by seasonal effects as baseline data were collected in autumn whereas data from the first lockdown were collected in June 2020. A recent study on sleep timing in students in the city of Seattle during all four seasons showed that chronotype was delayed by about 30 minutes in autumn/winter in comparison to summer [47]. Therefore, a delay in chronotype caused by the lockdown in spring 2020 might be masked by a seasonal shift to an earlier chronotype in spring/summer times in our students as well.

Strengths of our study include detailed determination of body composition by BIA at baseline. Unfortunately, we were not able to reanalyse body composition since the university was closed at the time of our online survey. We assessed both pre- and lockdown data prospectively using the exact same questionnaires. The fact that pre-lockdown data were assessed shortly before the first lockdown in March 2020 allows to determine the specific impact of the lockdown on circadian data and physical activity behaviour.

In conclusion, our data show that a later chronotype and a higher SJL are of selective relevance for a higher visceral fat mass also among relatively healthy young adults. Physical activity behaviour or the relevance given to physical activity accounts for the association of chronotype with visceral fat mass. Therefore, as stated by others, chronobiology should be considered in the development of personalized health strategies [11,35], in particular specific strategies might be necessary to assist later chronotypes with the implementation of a healthy life style. Further studies are necessary to explore motivators promoting attention to physical activity in a chronotype-specific manner and whether the preference for physical activity behaviour or even the efficacy of different physical activity schedules can be adapted to the individual circadian rhythm.

## Supporting information

S1 Data (DOCX)

## References

[1] Roenneberg T, Wirz-Justice A, Merrow M. Life between clocks: daily temporal patterns of human chronotypes. J Biol Rhythms. 2003;18:80–90. doi: 10.1177/0748730402239679 12568247

[2] Roenneberg T, Merrow M. The Circadian Clock and Human Health. Curr Biol. 2016;26:R432–43. doi: 10.1016/j.cub.2016.04.011 27218855

[3] Roenneberg T, Kuehnle T, Juda M, Kantermann T, Allebrandt K, Gordijn M, et al. Epidemiology of the human circadian clock. Sleep Med Rev. 2007;11:429–38. doi: 10.1016/j.smrv.2007.07.005 17936039

[4] Rothman KJ. BMI-related errors in the measurement of obesity. Int J Obes (Lond). 2008;32 Suppl 3:S56–9. doi: 10.1038/ijo.2008.87 18695655

[5] de Amicis R, Galasso L, Leone A, Vignati L, de Carlo G, Foppiani A, et al. Is Abdominal Fat Distribution Associated with Chronotype in Adults Independently of Lifestyle Factors? Nutrients 2020. doi: 10.3390/nu12030592 32106417 PMC7146439

[6] McMahon DM, Burch JB, Youngstedt SD, Wirth MD, Hardin JW, Hurley TG, et al. Relationships between chronotype, social jetlag, sleep, obesity and blood pressure in healthy young adults. Chronobiol Int. 2019;36:493–509. doi: 10.1080/07420528.2018.1563094 30663440

[7] Wennman H, Kronholm E, Partonen T, Peltonen M, Vasankari T, Borodulin K. Evening typology and morning tiredness associates with low leisure time physical activity and high sitting. Chronobiol Int. 2015;32:1090–100. doi: 10.3109/07420528.2015.1063061 26317556

[8] Mota MC, Waterhouse J, De-Souza DA, Rossato LT, Silva CM, Araújo MBJ, et al. Association between chronotype, food intake and physical activity in medical residents. Chronobiol Int. 2016;33:730–9. doi: 10.3109/07420528.2016.1167711 27096153

[9] Lavie CJ, Ozemek C, Carbone S, Katzmarzyk PT, Blair SN. Sedentary Behavior, Exercise, and Cardiovascular Health. Circ Res. 2019;124:799–815. doi: 10.1161/CIRCRESAHA.118.312669 30817262

[10] Khoury SR, Evans NS, Ratchford EV. Exercise as medicine. Vasc Med. 2019;24:371–4. doi: 10.1177/1358863X19850316 31144595

[11] Vitale JA, Weydahl A. Chronotype, Physical Activity, and Sport Performance: A Systematic Review. Sports Med. 2017;47:1859–68. doi: 10.1007/s40279-017-0741-z 28493061

[12] Facer-Childs E, Brandstaetter R. The impact of circadian phenotype and time since awakening on diurnal performance in athletes. Curr Biol. 2015;25:518–22. doi: 10.1016/j.cub.2014.12.036 25639241

[13] Erren TC, Lewis P. SARS-CoV-2/COVID-19 and physical distancing: risk for circadian rhythm dysregulation, advice to alleviate it, and natural experiment research opportunities. Chronobiol Int. 2020;37:1106–9. doi: 10.1080/07420528.2020.1772811 32498625

[14] Korman M, Tkachev V, Reis C, Komada Y, Kitamura S, Gubin D, et al. COVID-19-mandated social restrictions unveil the impact of social time pressure on sleep and body clock. Sci Rep. 2020;10:22225. doi: 10.1038/s41598-020-79299-7 33335241 PMC7746700

[15] Staller N, Randler C. Changes in sleep schedule and chronotype due to COVID-19 restrictions and home office. Somnologie (Berl). 2020:1–7. doi: 10.1007/s11818-020-00277-2 33223953 PMC7670483

[16] Gallè F, Sabella EA, Ferracuti S, de Giglio O, Caggiano G, Protano C, et al. Sedentary Behaviors and Physical Activity of Italian Undergraduate Students during Lockdown at the Time of CoViD-19 Pandemic. Int J Environ Res Public Health 2020. doi: 10.3390/ijerph17176171 32854414 PMC7504707

[17] Luciano F, Cenacchi V, Vegro V, Pavei G. COVID-19 lockdown: Physical activity, sedentary behaviour and sleep in Italian medicine students. Eur J Sport Sci. 2020:1–10. doi: 10.1080/17461391.2020.1842910 33108970

[18] Tavolacci MP, Wouters E, van de Velde S, Buffel V, Déchelotte P, van Hal G, et al. The Impact of COVID-19 Lockdown on Health Behaviors among Students of a French University. Int J Environ Res Public Health 2021. doi: 10.3390/ijerph18084346 33923943 PMC8072635

[19] Bertrand L, Shaw KA, Ko J, Deprez D, Chilibeck PD, Zello GA. The impact of the coronavirus disease 2019 (COVID-19) pandemic on university students’ dietary intake, physical activity, and sedentary behaviour. Appl Physiol Nutr Metab. 2021;46:265–72. doi: 10.1139/apnm-2020-0990 33449864

[20] Romero-Blanco C, Rodríguez-Almagro J, Onieva-Zafra MD, Parra-Fernández ML, Del Prado-Laguna MC, Hernández-Martínez A. Physical Activity and Sedentary Lifestyle in University Students: Changes during Confinement Due to the COVID-19 Pandemic. Int J Environ Res Public Health 2020. doi: 10.3390/ijerph17186567 32916972 PMC7558021

[21] Gallego-Gómez JI, Campillo-Cano M, Carrión-Martínez A, Balanza S, Rodríguez-González-Moro MT, Simonelli-Muñoz AJ, et al. The COVID-19 Pandemic and Its Impact on Homebound Nursing Students. Int J Environ Res Public Health 2020. doi: 10.3390/ijerph17207383 33050435 PMC7600682

[22] Korman M, Tkachev V, Reis C, Komada Y, Kitamura S, Gubin D, et al. Outdoor daylight exposure and longer sleep promote wellbeing under COVID-19 mandated restrictions. J Sleep Res. 2022;31:e13471. doi: 10.1111/jsr.13471 34549481 PMC8646753

[23] Krug S, Jordan S, Mensink GBM, Müters S, Finger J, Lampert T. Körperliche Aktivität: Ergebnisse der Studie zur Gesundheit Erwachsener in Deutschland (DEGS1). [Physical activity: results of the German Health Interview and Examination Survey for Adults (DEGS1)]. Bundesgesundheitsblatt Gesundheitsforschung Gesundheitsschutz. 2013;56:765–71. doi: 10.1007/s00103-012-1661-6 23703496

[24] Mitteldeutscher Rundfunk, Chronik der Corona-Krise. https://www.mdr.de/nachrichten/jahresrueckblick/corona-nachrichten-jahresrueckblick-chronologie-100.html.

[25] Harris PA, Taylor R, Thielke R, Payne J, Gonzalez N, Conde JG. Research electronic data capture (REDCap)—a metadata-driven methodology and workflow process for providing translational research informatics support. J Biomed Inform. 2009;42:377–81. doi: 10.1016/j.jbi.2008.08.010 18929686 PMC2700030

[26] Roenneberg T, Wirz-Justice A, Merrow M. Life between clocks: daily temporal patterns of human chronotypes. J Biol Rhythms. 2003;18:80–90. doi: 10.1177/0748730402239679 12568247

[27] Roenneberg T, Pilz LK, Zerbini G, Winnebeck EC. Chronotype and Social Jetlag: A (Self-) Critical Review. Biology (Basel) 2019. doi: 10.3390/biology8030054 31336976 PMC6784249

[28] Bosy-Westphal A, Schautz B, Later W, Kehayias JJ, Gallagher D, et al. What makes a BIA equation unique? Validity of eight-electrode multifrequency BIA to estimate body composition in a healthy adult population. Eur J Clin Nutr. 2013;67 Suppl 1:S14–21. doi: 10.1038/ejcn.2012.160 23299866

[29] T. Lohman, A. Roache, R. Martorell. Anthropometric Standardization Reference Manual. In; 1988.

[30] Maldonado G, Greenland S. Simulation study of confounder-selection strategies. Am J Epidemiol. 1993;138:923–36. doi: 10.1093/oxfordjournals.aje.a116813 8256780

[31] Garaulet M, Gómez-Abellán P, Alburquerque-Béjar JJ, Lee Y-C, Ordovás JM, Scheer FAJL. Timing of food intake predicts weight loss effectiveness. Int J Obes (Lond). 2013;37:604–11. doi: 10.1038/ijo.2012.229 23357955 PMC3756673

[32] Roenneberg T, Allebrandt KV, Merrow M, Vetter C. Social jetlag and obesity. Curr Biol. 2012;22:939–43. doi: 10.1016/j.cub.2012.03.038 22578422

[33] Parsons MJ, Moffitt TE, Gregory AM, Goldman-Mellor S, Nolan PM, Poulton R, et al. Social jetlag, obesity and metabolic disorder: investigation in a cohort study. Int J Obes (Lond). 2015;39:842–8. doi: 10.1038/ijo.2014.201 25601363 PMC4422765

[34] Vetrani C, Barrea L, Verde L, Sarno G, Docimo A, de Alteriis G, et al. Evening chronotype is associated with severe NAFLD in obesity. Int J Obes (Lond). 2022;46:1638–43. doi: 10.1038/s41366-022-01159-3 35676442

[35] Adan A, Archer SN, Hidalgo MP, Di Milia L, Natale V, Randler C. Circadian typology: a comprehensive review. Chronobiol Int. 2012;29:1153–75. doi: 10.3109/07420528.2012.719971 23004349

[36] Rahafar A, Castellana I, Randler C, Antúnez JM. Conscientiousness but not agreeableness mediates females’ tendency toward being a morning person. Scand J Psychol. 2017;58:249–53. doi: 10.1111/sjop.12362 28543321

[37] Lenneis A, Vainik U, Teder-Laving M, Ausmees L, Lemola S, Allik J, et al. Personality traits relate to chronotype at both the phenotypic and genetic level. J Pers. 2021;89:1206–22. doi: 10.1111/jopy.12645 33998684

[38] Zerón-Rugerio MF, Hernáez Á, Porras-Loaiza AP, Cambras T, Izquierdo-Pulido M. Eating Jet Lag: A Marker of the Variability in Meal Timing and Its Association with Body Mass Index. Nutrients 2019. doi: 10.3390/nu11122980 31817568 PMC6950551

[39] Ayala V, Martínez-Bebia M, Latorre JA, Gimenez-Blasi N, Jimenez-Casquet MJ, Conde-Pipo J, et al. Influence of circadian rhythms on sports performance. Chronobiol Int. 2021;38:1522–36. doi: 10.1080/07420528.2021.1933003 34060402

[40] Marinac CR, Quante M, Mariani S, Weng J, Redline S, Cespedes Feliciano EM, et al. Associations Between Timing of Meals, Physical Activity, Light Exposure, and Sleep With Body Mass Index in Free-Living Adults. Journal of Physical Activity and Health. 2019;16:214–21. doi: 10.1123/jpah.2017-0389 30798690 PMC6440200

[41] Hasan MM, Jankowski KS, Khan MHA. Morningness-eveningness preference and shift in chronotype during COVID-19 as predictors of mood and well-being in university students. Pers Individ Dif. 2022;191:111581. doi: 10.1016/j.paid.2022.111581 35250137 PMC8882407

[42] Leone MJ, Sigman M, Golombek DA. Effects of lockdown on human sleep and chronotype during the COVID-19 pandemic. Curr Biol. 2020;30:R930–R931. doi: 10.1016/j.cub.2020.07.015 32810450 PMC7342078

[43] Oved S, Mofaz M, Lan A, Einat H, Kronfeld-Schor N, Yamin D, et al. Differential effects of COVID-19 lockdowns on well-being: interaction between age, gender and chronotype. J R Soc Interface. 2021;18:20210078. doi: 10.1098/rsif.2021.0078 34062107 PMC8169206

[44] Salfi F, Amicucci G, Corigliano D, D’Atri A, Viselli L, Tempesta D, et al. Changes of evening exposure to electronic devices during the COVID-19 lockdown affect the time course of sleep disturbances. Sleep 2021. doi: 10.1093/sleep/zsab080 34037792 PMC8194574

[45] Allebrandt KV, Teder-Laving M, Kantermann T, Peters A, Campbell H, Rudan I, et al. Chronotype and sleep duration: the influence of season of assessment. Chronobiol Int. 2014;31:731–40. doi: 10.3109/07420528.2014.901347 24679223

[46] Tucker P, Gilliland J. The effect of season and weather on physical activity: a systematic review. Public Health. 2007;121:909–22. doi: 10.1016/j.puhe.2007.04.009 17920646

[47] Dunster GP, Hua IJ, Grahe A, Fleischer JG, Panda S, Wright KP, et al. Daytime light exposure is a strong predictor of seasonal variation in sleep and circadian timing of university students. J Pineal Res 2022. doi: 10.1111/jpi.12843 36404490

