# Supplementary material for: The association of chronotype and social jet lag with body composition in German students: The role of physical activity behaviour and the impact of the pandemic lockdown
Source: PLoS One. 2023 Jan 11;18(1):e0279620. doi: 10.1371/journal.pone.0279620 (PMC9833523; doi:10.1371/journal.pone.0279620)
Supplement: S1 Data — (DOCX) [file pone.0279620.s001.docx]

**Supplemental data**

**Table S: Comparison of baseline characteristics of students participating in the follow-up online survey (n=156) and students which did not participate (n=164)**

|  | Characteristics of participants | Characteristics of non-participants |
| --- | --- | --- |
| Sex n (% females) | 104 (67) | 80 (49) |
| Age (years) | 22.9 (2.0) | 22.8 (1.9) |
| BMI (kg/m^2^) | 22.8 (2.9) | 23.4 (3.1) |
| Smokers n (%) | 11 (7) | 21 (13) |
|  |  |  |
| *Body composition* |  |  |
| Visceral fat mass (kg) |  |  |
| Males | 0.62 (0.21; 1.1) | 0.72 (0.33; 1.2) |
| Females | 0.37 (0.22; 0.6) | 0.35 (0.17; 0.58) |
| Skeletal muscle mass (kg) |  |  |
| Males | 31.5 (28.2; 34.6) | 32.1 (27.0; 35.1) |
| Females | 20.2 (18.9; 22.1) | 20.4 (18.8; 22.4) |
| FMI (kg/m^2^) |  |  |
| Males | 3.3 (2.7; 5.3) | 4.1 (3.0; 5.1) |
| Females | 6.0 (4.9; 7.3) | 5.6 (4.7; 7.5) |
| FFMI (kg/m^2^) |  |  |
| Males | 19.3 (18.1; 20.7) | 20.1 (18.8; 21.1) |
| Females | 16.1 (15.3; 17.2) | 16.2 (15.5; 17.0) |
|  |  |  |
| *Chronotype and social jet lag* |  |  |
| Chronotype (h:mm) a.m. | 4:30 (3:43; 5:21) | 4:38 (4:00; 5:15) |
| Social jet lag (h:mm) | 1:07 (0:35; 1:45) | 1:05 (0:45; 1:45) |
|  |  |  |
| PA ≥ 150 min/week ** | 133 (85) | 34 (21) |
|  |  |  |
| Attention to PA:  “important/very important” n (%) | 80 (52) | 91 (56) |

Abbreviations: BMI - body mass index, FMI – fat mass index, FFMI – fat free mass index, n – sample size, smoker (yes/no), job (yes/no), PA – physical activity

Data are frequencies, mean (SD) or medians (Q1; Q3).

* physical activity refers to all kind of exhaustive physical activity including exercise frequency and types of exercise were questioned additionally.

** high physical activity means > 150 minutes/week according to the WHO recommendations (https://www.who.int/news-room/fact-sheets/detail/physical-activity).

***multiple answers were possible.
